# Anti-Tumor Effects of Exosomes Derived from Drug-Incubated Permanently Growing Human MSC

**DOI:** 10.3390/ijms21197311

**Published:** 2020-10-03

**Authors:** Catharina Melzer, Juliane von der Ohe, Ralf Hass

**Affiliations:** Biochemistry and Tumor Biology Lab, Department of Obstetrics and Gynecology, Hannover Medical School, 30625 Hannover, Germany; catharina.melzer@t-online.de (C.M.); Ohe.Juliane.von.der@mh-hannover.de (J.v.d.O.)

**Keywords:** mesenchymal stroma/stem-like cells, tumor microenvironment, cell interaction, exosomes, tumor therapy

## Abstract

Similar to growth-limited human primary cultures of mesenchymal stroma/stem-like cells (MSC), the continuously proliferating human MSC544 cell line produced extracellular vesicles as characterized by expression of the tetraspanin molecules CD9, CD63, and CD81. Release of these particles was predominantly detectable during continuous cell growth of MSC544 in contrast to confluency-mediated transient growth arrest. For therapeutic use, these particles were isolated from proliferating MSC544 after taxol treatment and applied to different cancer cell cultures. A pronounced cytotoxicity of lung, ovarian, and breast cancer cells was observed primarily with taxol-loaded exosomes, similar to the effects displayed by application of taxol substance. While these findings suggested pronounced cancer cell targeting of MSC544 exosomes, a tumor therapeutic approach was performed using a mouse in vivo breast cancer model. Thus, intravenous injection of taxol-loaded MSC544 exosomes displayed superior tumor-reducing capabilities as compared to application of taxol exosomes by oral gavage. To broaden this therapeutic spectrum, epirubicin was applied to MSC544, and the derived exosomes likewise exhibited significant cytotoxic effects in different cancer cell cultures. These findings suggest an unlimited source for large-scale exosome production with reproducible quality to enable variable drug targeting of tumors or other diseases.

## 1. Introduction

Various clinical studies are using mesenchymal stroma/stem-like cells (MSC) for different clinical approaches, including hematological disease, graft-versus-host disease, organ transplantation, diabetes, inflammatory diseases, bone and cartilage, neurological, and skin diseases, among others [1,2,3,4]. However, the use of human MSC for tissue replacement therapies or regenerative medicine is limited by eventual growth arrest and senescence due to a finite life span of primary MSC. This requires expansion of frequently new MSC populations and complicates reliability and reproducibility of the stem cell source, e.g., by donor heterogeneity. Thus, standardization with a permanently proliferating human MSC cell line model, such as MSC544, provides a unique cell source and reduces heterogeneity for potential clinical utilization [5].

MSC per se already represent a heterogeneous population, also termed multipotent mesenchymal stromal cells or medicinal signaling cells [6,7]. Substrate adherence, migratory activity, differentiation capacity at least along mesenchymal phenotypes, and distinct core surface marker expression, such as CD73, CD90, and CD105, with simultaneous absence of at least CD14, CD31, CD34, and CD45 represent minimal characteristics of MSC as stromal cells. Other marker expression or stem-like properties, such as self-renewal capacity, may only apply to a small subset within an MSC population which comprises different subpopulations. Accordingly, MSC heterogeneity is displayed by small subpopulations of stem-like cells and further special properties of other stromal subpopulations lacking stem-like features but displaying the minimal MSC characteristics which can be maintained during prolonged in vitro culture [8]. Moreover, these minimal characteristics are also shared by more differentiated populations, such as pericytes and fibroblasts. However, the characteristics of MSC vary upon culture conditions and a changing environment by diverse stimuli in vitro, and may be different when compared to the corresponding tissue-originating MSC populations in vivo [2,9].

MSC are predominantly found in perivascular regions of various adult organs and tissues [10,11]. In addition, human neonatal tissues, such as amniotic and chorionic membranes, decidua, whole placenta, cord blood, and umbilical cord (hUC), provide a non-invasive and ethically non-problematic MSC source with superior growth and expansion capacity [12].

Besides the use of MSC as a transplantable cell system, release of extracellular vesicles (EVs) as cell-free vesicular products, including microvesicles or MSC-derived exosomes, is gaining increased attention for potential clinical applications [13]. Exosomes represent small particles, approximately 20 to 200nm in diameter, as multivesicular bodies of endocytic origin, released into the extracellular compartment [14]. These particles contain a variety of different proteins, DNA, mRNAs, regulatory microRNAs (miRs), long non-coding RNAs, and circular RNAs, among other signaling molecules which can alter the functionality of recipient cells [15,16]. Exosomes can be identified by typical marker proteins, including surface glycoproteins of the tetraspanin transmembrane-4 family, such as CD9, CD63, and CD81 (= TAPA-1 (target of the antiproliferative antibody 1) = tetraspanin-28) [15,17].

A variety of different cells release exosomes, including populations of a tumor microenvironment, such as endothelial cells, immune cells, MSC, and cancer cells. Thus, cancer cell-derived exosomes contain many different proteins and tumor-specific nucleic acids which are transmitted and incorporated by nearby residing cells, such as MSC. As a consequence, MSC can be re-programmed by conversion of their normal trophic properties into tumor-supportive functionalities. Conversely, uptake of MSC-released exosomes by cancer cells can change the tumor functionality of several different tumor types, including breast and ovarian cancers [18]. Thereby, MSC-derived exosomes can confer signals for inhibition or promotion of tumor growth. Accordingly, mutual exchange of exosomes between MSC and cancer cells contributes to alter cancer cell functionalities and vice versa to modify MSC into carcinoma-associated (CA)-MSC, eventually differentiating into carcinoma-associated fibroblasts (CAFs) [19,20,21,22]. These findings indicate that the exosomal content displays large variability according to the different exosome-secreting cell populations. Moreover, exosomes significantly change in disease, inflammation, and cancer [2]. Although some controversy still exists, the potential dualism of the tumor-inhibitory and tumor-promoting effects of MSC-derived exosomes can be attributed predominantly to the MSC source and the microenvironment. In particular, tumor-unrelated MSC exosomes, for example, may suppress angiogenesis and relay signals for dormancy of breast cancer by shuttling miR-100, miR-16, miR-23b, and miR-222/223. In contrast, tumor-related exosomes are considered to be closely associated with the pathogenesis and microenvironmental development of cancers. Consequently, exosomes released by CA-MSC or CAFs can promote tumor growth and cancer cell migration in different tumors, such as gastric tumors, via activation of Akt, ERK1/2, and miR-221, or in breast cancers, by delivering exosomal TGF-beta, semaphorins, MMP-2, miR-21, and miR-34a, among others [23].

Loading of exosomes with cargo and transport to appropriate target cells represents useful non-cellular therapeutic vehicles to address, for example, primary and metastatic cancer cells. Moreover, treatment of cancers with different chemotherapeutic agents frequently is associated with severe side effects. In certain cases, particularly by using alkylating agents, platinum-based drugs, or anthracycline topoisomerase II inhibitors, this collateral damage can even induce secondary cancerous diseases, such as myelodysplastic syndrome and acute myelogenous leukemia [24]. Therefore, the present study examined a potential release of EVs from the permanently growing human MSC544 cell line. Furthermore, this work introduced the use of drug-loaded exosomes from MSC544 as a constant cell source for obtaining appropriate quantities in a reproducible cellular model to enable more specific tumor targeting.

## 2. Results

### 2.1. Characterisation of MSC544-Derived Microvesicles and Exosomes

**Figure 1:**
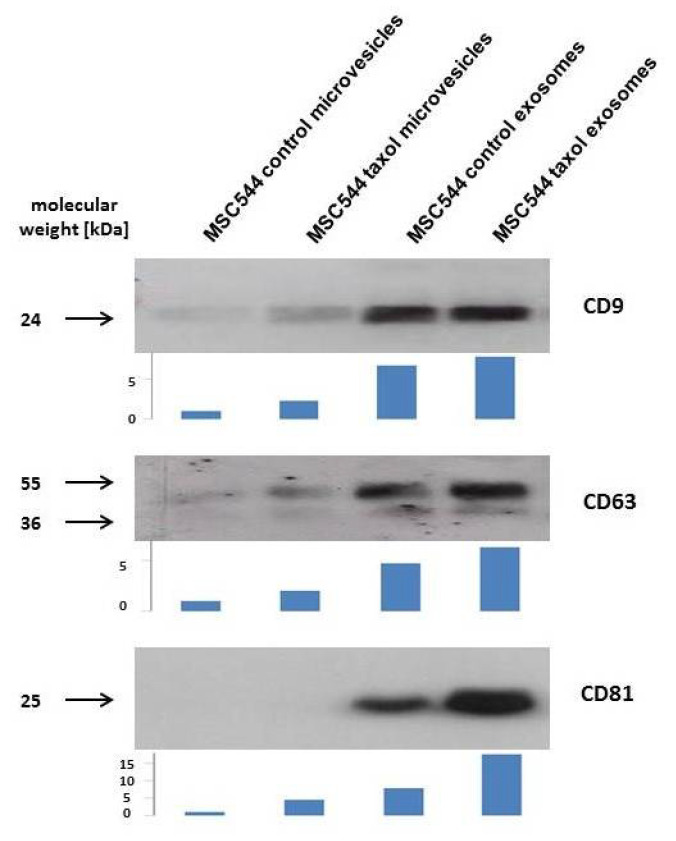
Following vesicle preparation of continuously growing control MSC544 and previously taxol-treated MSC544, the precipitated vesicles of the 10,000× *g* centrifugation were used as microvesicles and the 100,000 × *g* centrifugation was used as exosome fraction according to previous characterizations [28]. Vesicles were homogenized and protein was measured by BCA method. Aliquots of 10 µg protein/lane were analyzed by Western blot for expression of exosome-related tetraspanins. Quantification of the Western blots was performed by densitometry scanning using the image J software. Arbitrary units of each scan are demonstrated by bar size below the blots.

Previous work has demonstrated that a variety of different MSC from primary cultures exhibit mutual interactions with cancer cells of breast and ovarian tumors in vitro and in vivo [25,26,27]. In addition to direct cell-to-cell contacts, further cellular communication was performed via indirect mechanisms by release of biological factors and/or small vesicles. Similar observations were obtained during co-culture of the permanently growing human MSC-like cell line MSC544 with the triple negative human breast cancer cell line MDA-MB-231. GFP-labeled MSC544 and cherry-labeled MDA-MB-231 cells mutually exchanged EVs, including microvesicles and exosomes. While release of MSC544 EVs was addressed to the breast cancer cells, it was of interest to further separate these vesicles with respect to a potentially therapeutic use from an unlimited and reproducible cell source. Thus, a previous mouse model suggested an advantage of using taxol-loaded exosomes from primary MSC for targeting metastatic breast cancer [28]. Accordingly, MSC544 were similarly treated with 10 µM taxol for 24 h and microvesicles (10,000× *g* fraction) and exosomes (100,000× *g* fraction) were isolated by sequential centrifugation of a conditioned, serum-free MSC544 medium after 24 h. Typical protein markers of exosomes include at least surface glycoproteins of the tetraspanin transmembrane-4 family, such as CD9, CD63, and CD81 (=TAPA-1 (target of the antiproliferative antibody 1) = tetraspanin-28) [15,17]. Corresponding Western blot analysis of the MSC544-derived vesicles revealed little presence of CD9 and CD63 in the control and taxol microvesicle preparations, whereby CD81 was barely detectable. In contrast, all three types of tetraspanin molecules were markedly expressed in the two exosome preparations from the MSC544 control and taxol-treated cells (Figure 1).

These findings demonstrated that permanently growing MSC544 cells produce and release EVs. Moreover, exosomes can be enriched from the crude microvesicle fraction with appropriate markers carrying the typical tetraspanin molecules.

**Figure 2:**
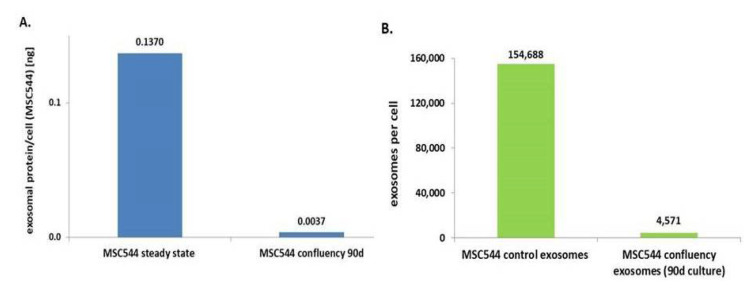
(**A**) Exosomes from continuously growing MSC544 and from growth-arrested cells after 90 days of continuously confluent culture were isolated and protein concentrations were determined and normalized to the cell number by calculation of the amount of exosomal protein per cell. (**B**) Measurement of the number of isolated exosomes from continuously growing MSC544 and from 90-day growth-arrested cells after 24 h in serum-free culture medium was performed by nanoparticle tracking analysis (NTA) in a ZetaView PMX120. The number of exosomes per cell was calculated following cell counting of the corresponding MSC544 cultures by trypan blue exclusion using a hemocytometer.

Previous work has demonstrated that MSC544 can undergo transient cell cycle arrest during confluency. These changes from a proliferative to a growth-inhibited and senescent-like state of MSC544 cultures display reversible functional alterations associated with marked differences in metabolic activities [5]. Accordingly, we were interested to test presumably altered exosome production during this phase of confluency-mediated growth arrest. Potential differences in exosome production of continuously growing or growth-arrested confluent MSC544, for example, provide important information about the preferred exosome source depending on the cellular conditions of MSC544. Therefore, exosomes from continuously growing MSC544 and from 90-day growth-inhibited confluent MSC544 were isolated and quantified for protein content of the exosomal fraction. A comparison of the two MSC544 cultures revealed about a 37-fold increased amount of exosomal protein per cell in the proliferating cultures in contrast to growth-arrested MSC544 (Figure 2A). These findings were substantiated by nanoparticle tracking analysis (NTA) light scatter measurements of exosome preparations from these two subpopulations. Thus, continuously growing MSC544 cultures generated about 1.55 × 10^5^ exosomes per cell per 24 h, which is in a similar range compared to the exosome production by different primary UC-MSC cultures [28]. In contrast, 90-day growth-inhibited confluent MSC544 released only about 4.57 × 10^3^ exosomes per cell into the serum-free culture medium after 24 h (Figure 2B). The ratio of these differences demonstrated about a 34-fold higher generation of exosomes in the proliferating MSC544 cultures as during growth-inhibited senescence-like state.

These findings suggested that exosome production and release was significantly enhanced by 34- to 37-fold in proliferating MSC544 in contrast to growth-arrested confluent MSC544 as evaluated by two different and independent methods. Consequently, continuously growing MSC544 represent the preferred source for isolation of large exosome quantities.

### 2.2. In Vitro Anti-Tumor Activity of MSC544-Derived Microvesicles and Exosomes

**Figure 3:**
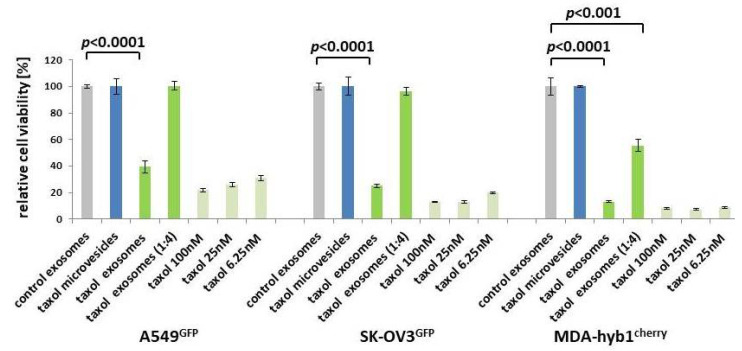
Microvesicles and exosomes were isolated from continuously proliferating control MSC544 and 24 h taxol-treated MSC544. Control exosomes (grey bars) were handled as a reference with 100% viability and compared to taxol-loaded microvesicles (blue bars), to taxol-loaded exosomes together with a 1:4 dilution (green bars), and to different concentrations of taxol substance (light green bars). The different stimuli were tested after a 72-h incubation of GFP- or cherry-labeled lung (A549^GFP^), ovarian (SK-OV3^GFP^), and breast (MDA-hyb1^cherry^) cancer cells. Each set of data represents the mean ± s.d. of three independent experiments and significance was calculated by unpaired Student’s *t*-test.

Further tests were performed with respect to a potential tumor-targeting bioactivity of the different vesicles isolated from proliferating MSC544 with and without taxol-treatment. Thus, microvesicles and exosomes were isolated from subsequent 24 h serum-free medium supernatants and analyzed by NTA (Table 1):

The negative surface charge as determined by the zeta potential at 25 °C confirmed a reasonable quality, mobility, and stability of the particle preparations due to a reduced tendency to aggregate and become more unstable [29]. Equal amounts of the vesicles were tested for cytotoxic effects in a fluoroscan assay following incubation of A549^GFP^ lung cancer, SK-OV3^GFP^ ovarian cancer, and MDA-hyb1^cherry^ breast cancer cells for 72 h. Compared to control exosomes with 100% viability, similar results were obtained with microvesicles from previously taxol-treated MSC544. In contrast, exosomes prepared from previously taxol-exposed MSC544 significantly reduced the viability of A549^GFP^ lung cancer cells by 60.6 ± 4.4%, of SK-OV3^GFP^ ovarian cancer cells by 75.1 ± 1.0%, and of MDA-hyb1^cherry^ breast cancer cells by 86.9 ± 0.7% (Figure 3). Moreover, a 1:4 dilution of taxol exosomes also significantly diminished MDA-hyb1^cherry^ breast cancer cell viability by 45.5 ± 4.6%. Treatment with different amounts of taxol substance demonstrated concentration-dependent cytotoxicity, whereby 6.25 nM taxol declined the viability of A549^GFP^ lung cancer cells by 68.9 ± 1.9%, that of SK-OV3^GFP^ ovarian cancer cells by 80.3 ± 0.5%, and that of MDA-hyb1^cherry^ breast cancer cells by 91.3 ± 0.4, displaying similar effects compared to MSC544-derived taxol exosomes (Figure 3).

Together, these data demonstrated that taxol-loaded microvesicles from MSC544 had no detectable effect on the viability in any of the different tumor cell lines tested. By contrast, exosomes isolated from taxol-treated MSC544 exhibited cancer cell cytotoxicity similar to taxol substance in all three different tumor cell populations. These findings suggested that MSC544-derived exosomes rather than microvesicles displayed cancer cell-specific targeting.

### 2.3. In Vivo Application of Taxol-Treated MSC544-Derived Exosomes

**Figure 4:**
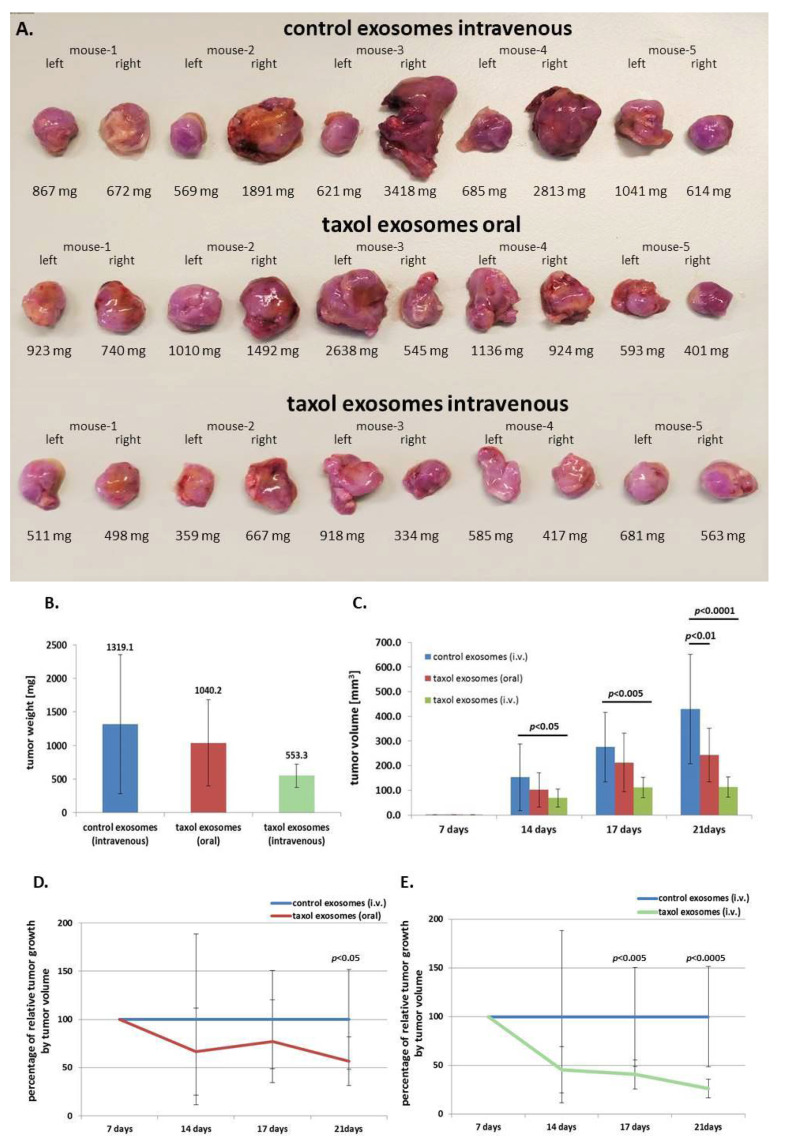
(**A**) The human triple negative breast cancer (TNBC)-derived hybrid cell line MDA-hyb1 was subcutaneously injected into the left and the right shoulders of 15 NOD/scid mice. Following the indicated exosomal treatments, the two-sided tumor development in all 15 mice was terminated after 21 days, whereby tumors were extracted and weighted. Moreover, distal organs were collected for analysis of metastases. (**B**) The average tumor weight was measured and calculated by combining the left and right tumors for each treatment group. Thus, data represent the mean ± s.d. of 10 tumors from five animals, respectively. (**C**) At the different treatment time points, tumor sizes were measured using a digital caliper. Corresponding volumes of MDA-hyb1 tumors were calculated with the longitudinal diameter (length) and the transverse diameter (width) in the modified ellipsoidal formula *V* = π/6 * width * (length)^2^ as previously reported [31,32]. (**D**) Differences in tumor volume of oral gavage of taxol exosomes and (**E**) differences in tumor volume of intravenous (i.v.) taxol exosomes at the different treatment time points were calculated as the percentage of control tumors (set to 100%) following application of control MSC544 exosomes.

To translate these findings into an in vivo model, subcutaneous tumors were induced in 15 NOD/scid mice by subcutaneous injection of 10^6^ human MDA-hyb1 triple negative breast cancer (TNBC) cells into the right and the left shoulder. After randomization of tumor-bearing mice, within 4 d of cancer cell application, treatment was started in three groups with five animals each until day 21 when all 30 tumors were dissected and weighted (Figure 4A). The three treatment groups included (a) intravenous injection of control exosomes, (b) oral gavage of taxol exosomes, and (c) intravenous injection of taxol exosomes from MSC544. Previous work demonstrated tumor-reduction of taxol-loaded milk-derived exosomes which were orally applied to target lung tumor xenografts [30]. Exosome treatment was performed at day 7, day 10, day 14, and day 17. Whereas oral gavage of taxol exosomes reduced the tumor weight by 21.1%, intravenous injection of MSC544-derived taxol exosomes was associated with a tumor weight decline of 58.1% (Figure 4B). A more pronounced effectiveness of MSC544-derived taxol exosomes became obvious by considering the tumor volume. The average control tumor volume of 430 ± 221 mm^3^ decreased by 43% to 244 ± 109 mm^3^ in oral gavage-applied MSC544 taxol exosome-treated tumors and was reduced by 74% (113 ± 41 mm^3^) following intravenously injected MSC544 taxol exosomes. These effects of tumor volume reduction by intravenous application of MSC544-derived taxol exosomes were also more pronounced as compared to a 63.9% tumor reduction previously observed with four primary MSC populations [28]. Calculations of the relative effectiveness of intravenously applied taxol substance reached a 97% reduction in tumor volume (Figure S1), which is identical to previous studies [28].

Following a time course of tumor volume measurement and calculations, a progressive decline in tumor size was observed for both application methods with taxol exosomes, whereby oral gavage of taxol exosomes reached a significant level after four treatment points at 21 days (Figure 4C,D). This tumor therapeutic effect was much more pronounced by intravenous injection of MSC544-derived taxol exosomes, achieving a significantly reduced tumor size already at the third treatment round after 17 d (Figure 4C,E).

**Figure 5:**
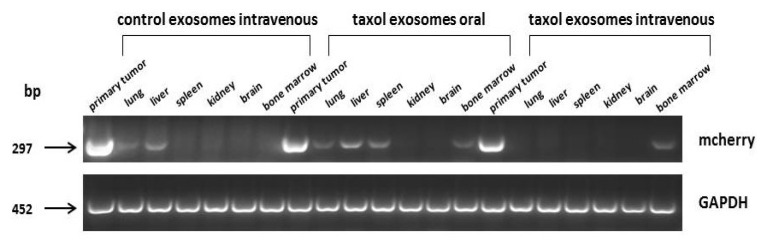
Expression of the MDA-hyb1 breast cancer cell-associated mCherry gene was analyzed by RT-PCR in the primary tumors and in distal organs. Equal loading of PCR products was documented by GAPDH transcripts.

Similar effects were observed during analysis of organ metastases. Expression of the mCherry gene, which was exclusively associated with the MDA-hyb1 breast cancer cells, was detectable in the lungs, liver, spleen, and bone marrow (Figure 5). Oral application of taxol-loaded exosomes displayed little, if any, effects when compared to intravenously injected control exosomes. In contrast, administration of a similar amount of MSC544-derived taxol exosomes displayed a markedly diminished mCherry expression in the primary tumor and in all organs except for the bone marrow (Figure 5).

In sum, systemic application of taxol-loaded exosomes from MSC544 exhibited in vivo anti-tumor effects by reduction of both the primary tumor and various distal organ metastases.

### 2.4. Further Drug Administration to MSC544-Derived Exosomes

**Figure 6:**
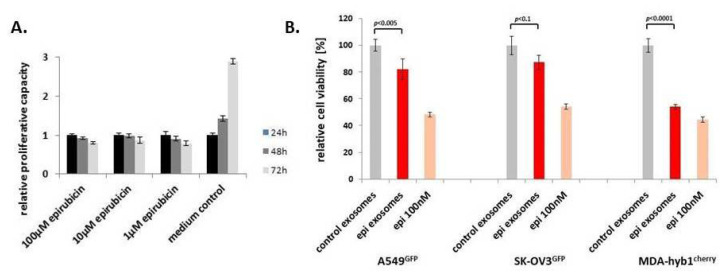
(**A**) Continuously growing MSC544^GFP^ were incubated with different concentrations of epirubicin for 24–72 h and the relative proliferative capacity was evaluated by fluoroscan assay, whereby the relative proliferation rate of control cells (medium control) was set to 1 as a reference. Data represent the mean ± s.d. (*n* = 10). (**B**) Exosomes were prepared from continuously growing control MSC544 and 10 µM epirubicin-treated MSC544 for 24 h. Similar aliquots of control and epirubicin-treated MSC544-derived (epi) exosomes were tested after a 72-h incubation of GFP- or cherry-labeled lung (A549^GFP^), ovarian (SK-OV3^GFP^), or breast (MDA-hyb1^cherry^) cancer cells. Relative cell viability was set to 100% for control exosomes (grey bars) and compared to epirubicin (epi) exosomes (red bars) and epirubicin (epi) substance (light red bars). Each set of data represents the mean ± s.d. of three independent experiments and significance was calculated by unpaired Student’s *t*-test.

To address the question as to whether MSC544 can also release exosomes loaded with other chemotherapeutic agents, we chose the anthrachinone derivative epirubicin, which is also frequently used as part of a therapeutic treatment in various cancer patients. First, potential cytotoxic effects of epirubicin on MSC544 were tested by application of different concentrations for 24 h to 72 h. Compared to the MSC544 culture in the control medium, incubation with up to 100 µM was associated with little, if any, differences in relative proliferative capacity within 24 h. Thereafter, all epirubicin treatments demonstrated a progressive decline in the MSC544 proliferation rate (Figure 6A). Based upon these results and previous findings with taxol-loaded exosomes, a comparable stimulation of MSC544 with 10 µM epirubicin for 24 h was chosen for the preparation of epirubicin-loaded exosomes. Quantification of the isolated particles from MSC544 by NTA revealed 0.9 × 10^12^ control exosomes/mL (average size 166 ± 77 nm) and 1.5 × 10^12^ epirubicin-loaded exosomes/mL (average size 179 ± 70 nm). Treatment of different cancer cell populations with equal amounts of epirubicin-loaded MSC544-derived exosomes compared to control exosomes demonstrated a significant cytotoxic effect on A549 lung cancer, SK-OV3 ovarian cancer, and MDA-hyb1 breast cancer cells, which was even more pronounced by application of 100 nM epirubicin substance (Figure 6B).

These findings with taxol and epirubicin suggest that different chemotherapeutic compounds in sublethal concentrations can be addressed by MSC544 cells to exosomes exhibiting tumor therapeutic effects.

## 3. Discussion

Exosomes isolated from different cell types, particularly from MSC, are discussed as a valuable tool as potential anti-tumor vehicles [2]. A cell-free clinical advantage of MSC-derived exosomes is also considering safety aspects as grafted cellular progenitors could raise some concerns in a clinical setting, such as aggregation and occlusion of small vessels [33], support of neoplasia [34], and pro-arrhythmic side problems [35]. MSC-derived exosomes are investigated in clinical trials among others for liver diseases [36], as well as for kidney, cardiovascular, and neurological diseases [37]. Moreover, exosomes obtained from MSC cultures are also considered in tumor therapeutic approaches, whereby these vesicles may confer signals for tumor-promoting and tumor-suppressing effects [38]. During mutual interactions and transmission of EVs and molecules within the tumor microenvironment, cancer cells, likewise, release exosomes containing specific mRNAs, microRNAs, and DNA fragments. Nearby residing cells, such as MSC, can incorporate these exosome-transmitted molecules by conversion into a tumor-promoting phenotype by induction of differentiation into CAFs (carcinoma-associated fibroblasts) which, in turn, can modulate cancer cells [39,40]. However, the variety of close interactions between MSC and cancer cells, such as exosome exchanges, provides an opportunity for specific tumor targeting [41]. Accordingly, MSC-derived exosomes can be educated by anti-tumor tools to selectively address and deliver therapeutic cargo to cancer cells. For example, genetically engineered MSC expressing TRAIL (tumor necrosis factor-related apoptosis-inducing ligand) can release TRAIL-loaded exosomes.

Another stimulation of MSC for TRAIL release and Dkk3 includes X-ray treatment [42,43]. As with X-rays, exposure of MSC to UV light [44] or heat [45] induces increased release of anti-tumor or immunogenic factors, such as TNF-α and IFN-γ. In addition, enhanced production of annexin A1 in radiotherapy-stimulated MSC can relay anti-tumor signals [46,47]. This may provide superior therapeutic effectiveness by a combined stimulation of MSC with radiotherapy and drugs. Furthermore, MSC can be activated by TNF-α to result in enhanced release of TRAIL and Dkk3 [48].

Isolation of TRAIL exosomes and application to tumor-bearing mice significantly reduced the tumor burden [49]. Exosomes derived from neonatal hUC-MSC have also emerged as potential therapeutic particles [50]. Thus, delivery of hUC-MSC exosomes loaded with exogenous miR-145-5p reduced invasion and proliferation of pancreatic ductal adenocarcinoma cells in vitro and also diminished tumor size in a mouse xenograft model in vivo [51]. Moreover, hUC-MSC transfected with miR-375 was associated with in vitro and in vivo inhibition of tumor growth and invasion of esophageal squamous cell carcinoma [52]. However, transfer of certain miRs can also neutralize effects such as the delivery of stroma-derived miR21 which mediates taxol resistance in ovarian cancer cells [53]. Despite these therapeutic approaches, clinical advantages are restricted by the finite life expectancy of primary MSC cultures which limits the availability of exosomes. Therefore, large quantities of MSC-derived exosomes for regenerative medicine or repetitive cancer treatment require exosome collection from multiple primary MSC cultures, raising problems of individual donor variability among others. Alternatively, permanent growth of human MSC-like cells may provide extracellular vesicles in desired quantities with appropriate reproducibility and standardized quality. Accordingly, different extracellular vesicles from MSC544 were tested.

MSC544 cease to grow after contact inhibition when the culture reaches confluency and develops a tissue-like structure [5]. Thereby, the cells enter a transient G0’-like arrest cycle, as previously suggested for a retrodifferentiation program and the role of protein kinase C signaling [54,55,56]. This halted proliferative capacity is associated with a senescence-like state by strong expression of senescence-associated beta-galactosidase and significantly altered metabolic activity of MSC544. The transient G0’-like cell cycle arrest can be maintained until the culture is placed again in a subconfluent environment. Thereby, MSC544 re-enter the proliferative cell cycle paralleled by immediate down-modulation of senescence-associated markers [5]. During such reversible growth arrest, however, a markedly reduced exosome production was detectable, suggesting that release of MSC544-generated exosomes is predominantly associated with progressive cell growth.

Consequently, different EVs from proliferating MSC544 were used for testing of potential anti-tumor activities. In contrast to taxol-loaded crude microvesicles displaying little, if any, therapeutic effects in lung, ovarian or breast cancer cultures in vitro, application of taxol-loaded exosomes was associated with significant cytotoxic activity similar to taxol substance in the various cancer cell types. As with MSC544, previous data in four primary human MSC populations demonstrated an analogous effectiveness using an equivalent amount of taxol-loaded exosomes. Of interest, these MSC-derived exosomes exhibited cytotoxic effects with about a 1000-fold reduced taxol concentration in exosomes when compared to application of pure taxol substance [28]. This suggested a more specific and effective cancer cell targeting by taxol-loaded MSC-derived exosomes. Moreover, exosome-encapsulated taxol may be better protected from metabolic degradation than the pure substance. Supportive evidence is obtained in taxol-treated breast cancer xenograft models in mice by the detection of a significant amount of taxol substance in the liver in contrast to taxol exosome-treated mice, suggesting constantly high levels of liver-associated taxol metabolization [28]. Therefore, exosome-protected taxol delivery may display longer lasting and more targeted effects as compared to the free chemotherapeutic substance.

The tumor-targeting efficiency of drug-loaded exosomes also depends on the application form. Indeed, predominant tumor reduction with exosomes from taxol-treated MSC544 was observed in primary tumors in vivo and accompanying distal organ metastases after intravenous injection rather than via oral gavage. These differences depending on the application method of exosomes also disclose the still scarce knowledge on pharmacokinetics and biodistribution of exosomes which requires further elucidation. Nevertheless, most types of exosomes from different cell sources achieved predominant physiological effects by systemic intravenous application [57].

With respect to the permanent proliferation capacity of MSC544, this provides a potentially unlimited exosome resource with reproducible quality. In addition, the selective effects of these exosomes to target primary tumors and metastases can contribute to reduce the suffering of patients from severe side effects caused by chemotherapeutic treatments. For example, taxol-mediated side effects can promote cardiotoxicity, myelosuppression, and neurotoxicity, with symptoms such as reduction in leukocyte numbers, hair loss (alopecia), painful muscles and joints, nausea and vomiting, skin reactions, diarrhea, and fatigue syndrome. Therefore, a major challenge of cancer chemotherapies is a concentrated addressing of nucleic acids or drugs into the tumor microenvironment with minimal collateral toxicity. A favorable approach is represented by nanodrug delivery systems via vehicles such as MSC-derived exosomes which can encapsulate these compounds and address the cargo, for example, to tumors, such as breast cancer cells. Moreover, MSC-derived exosomes can protect their cargo, such as taxol, against degradation [28] and facilitate their intracellular uptake via endocytosis [58,59]. These specific properties reduce adverse effects of chemotherapy and systemic toxicity.

A more tumor-specific addressing may be relevant particularly for cancer stem-like cells which can reside and develop in cancer stem cell niches [60] displaying special properties, such as self-renewal capacity for the maintenance of tumor growth, escape from immune surveillance, and resistance to various chemotherapeutic agents and to apoptosis.

MSC544-derived exosomes and their tropism towards different cancer cells as potential delivery vehicles was demonstrated besides taxol also for epirubicin by elevated cytotoxic activity in the different cancer cell lines. Thus, MSC544 exosomes can deliver several anti-cancer substances targeting different pathways. Taxol interferes with mitotic spindle assembly and chromosome segregation by stabilizing the microtubule polymer formation during cancer cell division. On the other hand, epirubicin acts at a completely different level by inhibition of topoisomerase II activity and intercalating between DNA base pairs to interfere with nucleic acid and protein synthesis. Although further substances still need to be tested, this suggested the capability of MSC544-derived exosomes to transmit a multi-modality compilation of anti-cancer drugs to tumors which underscores their therapeutic potential. A further advantage is displayed by the unlimited quantity and reproducible quality of MSC544-derived exosome production, representing an important prerequisite for prospective clinical usage.

## 4. Materials and Methods

### 4.1. Cell Culture

The human MSC544 cell line reflects a permanently growing MSC population as extensively characterized in previous work [5,61]. MSC544 were cultured in an MSC growth medium (αMEM (Sigma Chemie GmbH, Taufkirchen, Germany) supplemented with 10% allogeneic human AB-serum (blood from 31 male AB donors was commercially obtained from a blood bank at Hannover Medical School, Germany, and processed to a serum), 100 U/mL penicillin, 100 µg/mL streptomycin, and 2 mM L-glutamine (Sigma Chemie GmbH) at 37 °C with 5% CO_2_ in a humidified atmosphere. Subculture was performed following treatment of MSC544 by TrypLE (Life Technologies GmbH, Darmstadt, Germany) at 37 °C for 3 min.

Human MDA-hyb1 breast cancer cells represent an aggressive and highly metastatic breast cancer cell line originated after cell fusion of primary human MSC with the MDA-MB-231 breast cancer cell line [62]. MSC544 and MDA-hyb1 were cultured under xeno-free conditions in an MSC growth medium (αMEM (Sigma Chemie GmbH, Steinheim, Germany) supplemented with 10% allogeneic human AB-serum (blood from male AB donors was commercially obtained from a blood bank at Hannover Medical School, Germany, and processed to a serum), 100 U/mL penicillin, 100 µg/mL streptomycin, and 2 mM L-glutamine (Sigma Chemie GmbH, Taufkirchen, Germany). Subculture in passages (P) was performed following mechanical detachment of the loosely adherent MDA-hyb1 cells and by TrypLE (Life Technologies GmbH, Darmstadt, Germany) treatment of MSC544 at 37 °C for 3 min.

The human SK-OV-3 ovarian cancer cell line was commercially obtained in P25 from the ATCC, Manassas, VA, USA (ATCC^®^ #HTB-77TM). These cells were derived from the malignant ascites of a patient with progressive adenocarcinoma of the ovary. The human A549 lung carcinoma cell line was originally established from an explanted alveolar basal epithelial adenocarcinoma and cultured as previously described [63]. Both the ovarian and lung cancer cells were cultured at a density of 1750 cells/cm^2^ in RPMI 1640 supplemented with 10% (*v*/*v*) fetal calf serum, 2 mM L-glutamine, 100 U/mL penicillin, and 100 µg/mL streptomycin. Subculture was performed by trypsin/EDTA (Biochrom GmbH, Berlin, Germany) treatment for 5 min at 37 °C.

All cell lines were tested for mycoplasma by the luminometric MycoAlert Plus mycoplasma detection kit (Lonza Inc., Rockland, ME, USA) according to the manufacturer’s recommendations. Authentication of the different cell lines was performed by short tandem repeat (STR) fragment analysis using the GenomeLab human STR primer set (Beckman Coulter Inc., Fullerton, CA, USA). Identity of STR fragments was confirmed according to the STR database provided by the ATCC, Manassas, VA, USA, [32] and for MDA-hyb1 and MSC544 cell lines as outlined in previous work [5,62]. For measurement and quantification of the proliferation rate using the fluoroscan assay, the different cell populations were transduced with a 3rd generation lentiviral SIN vector containing the eGFP or the mCherry gene, respectively, as reported elsewhere [22].

### 4.2. Preparation and Analysis of MSC544-Derived Control Exosomes and Chemotherapeutic-Loaded Exosomes

Subconfluent MSC544 cultures (about 2 × 10^6^ cells) at a density of 1.4 × 10^4^ cells/cm^2^ were incubated in culture medium for 2 d. Thereafter, the culture medium was removed and the cells were washed three times with serum-free medium and then incubated with serum-free medium for 24 h. EVs (microvesicles and exosomes) were isolated following removal of the 24-h conditioned medium from the cells.

For chemotherapeutic loading, subconfluent MSC544 cultures were incubated with either an appropriate dilution (1:700) of solvent (50.17% ethanol = final concentration of 0.07% ethanol) for control exosomes or in the presence of 10 µM taxol (paclitaxel in solvent, diluted 1:700 from a 7 mM stock solution), or in the presence of 10 µM epirubicin for 24 h. The different MSC544 cultures were washed with a serum-free culture medium to remove non-incorporated drug and proteins. Thereafter, incubation was performed with a serum-free culture medium for a further 24 h and EVs were isolated from this 24-h conditioned medium.

While EV preparations represent a heterogenous mixture of small-sized extracellular vesicles, the present manuscript used the term ‘microvesicles’ and ‘exosomes’ for the secreted and isolated vesicles by MSC544, respectively. According to the protocol by Thery et al. [64] and the updated MISEV (minimal information for studies of extracellular vesicles) 2018 standards [65], preparation of MSC544-derived EVs was performed in four subsequent centrifugation steps (first step: 360× *g* for 10 min to remove cells; second step: 2000× *g* for 10 min to remove dead cells; third step: 10,000× *g* for 30 min to remove debris and large vesicles (microvesicles); fourth step: 100,000× *g* for 70 min to precipitate exosome-like particles) as extensively described previously [28].

Analysis and quantification of MSC544-derived EVs (microvesicles and exosomes) as well as therapeutic-loaded exosomes (taxol or epirubicin) for vesicle concentration, size distribution, and preparation quality was performed using the ZetaView PMX120 NTA (Particle Metrix GmbH, Meerbusch, Germany) with an embedded 40 mW laser at 488 nm and a CMOS camera. Protein aliquots of the exosomes were quantified using the colorimetric BCA assay (ThermoFisher Scientific, Schwerte, Germany). The isolated MSC544-derived EVs were resuspended in 50 µL of PBS and stored at −80 °C.

### 4.3. Immunoblot Analysis of Exosomes

Western blot analysis was performed as described previously [26]. Briefly, 10 µg aliquots of microvesicle protein or exosomal protein preparations from continuously growing control and taxol-treated MSC544, respectively, were separated on a 10% SDS polyacrylamide gel and transferred to a nitrocellulose membrane (GE Healthcare Lifescience, Freiburg, Germany) after semi-dry blotting (Peqlab Biotechnology GmbH, Erlangen, Germany) at 1.5 mA/cm^2^ for 1 h. The blots were incubated with a 1:500 dilution of the mouse monoclonal antibody CD9 (clone Ts9) (Invitrogen/ThermoFisher Scientific, Schwerte, Germany), a 1:250 dilution of the mouse monoclonal antibody CD63 (clone MX-49.129.5) (Santa Cruz Biotechnology, Inc., Heidelberg, Germany), and a 1:250 dilution of the mouse monoclonal antibody CD81 (clone M38) (Invitrogen//ThermoFisher Scientific, Schwerte, Germany).

### 4.4. In Vitro Cytotoxicity Measurements of Exosomes by Fluoroscan Assay

Several dilutions of control and taxol-loaded exosomes from MSC544 in the appropriate cell growth medium were applied to different cancer cell cultures. The proliferation rate was determined by fluorescence measurement using the fluoroscan assay as previously described [66]. Briefly, 1000 cells/well of A549^GFP^, SK-OV-3^GFP^, or MDA-hyb1^cherry^ populations were plated in flat-bottom 96-well plates (Nunc/ThermoFischer Scientific, Roskilde, Denmark) with (100 μL/well) standard culture medium. Following overnight attachment of the different cultures, 100 µL of drug solvent in culture medium was added as a control, and in further wells, 100 µL of culture medium containing appropriate dilutions of taxol or epirubicin substance, taxol- or epirubicin-loaded exosomes, and control exosomes was added to the cells. Following incubation for 72 h, the media were removed and the cells were lysed with 5% (*w*/*v*) SDS. Thereafter, the fluorescence intensities of GFP or cherry cell homogenates, which corresponded to the appropriate cell number of cancer cells, were measured at an excitation of 485 nm and an emission of 520 nm (GFP), or an excitation of 584 nm and an emission of 612 nm (cherry) using the Fluoroscan Ascent Fl (ThermoFisher Scientific, Schwerte, Germany).

### 4.5. In Vivo Experiments

Animal research using NOD/scid mice was performed by following the internationally recognized guidelines on animal welfare. The project has been approved by the institutional licensing committee ref. #33.19-42502-04-19/3080 on 2 July 2019.

About 10^6^ human MDA-hyb1 breast cancer cells were subcutaneously injected into the left and the right shoulders of fifteen 5–6-week-old female NOD/scid mice. Tumors smaller than 1mm^3^ were detectable after 4 days. All 15 tumor-bearing mice with 30 tumors were randomized into three treatment groups with 5 mice each:

Treatment 1: Intravenous application (100 µL) of MSC544-derived control exosomes.
Treatment 2: Oral gavage application (100 µL) of taxol-treated MSC544-derived exosomes. Oral application was performed using plastic feeding tubes (18 ga × 30 mm) (Instech Laboratories, Plymouth, PA, USA).
Treatment 3: Intravenous application (100 µL) of taxol-treated MSC544-derived exosomes

The different treatments were performed 4 times (at day 7, day 10, day 14, and day 17). Tumor progression was monitored and tumor size was measured using a digital caliper (VWR International, Darmstadt, Germany). At 21 days post-MDA-hyb1 cell transplantation, all animals were sacrificed by cervical dislocation when the tumor size of the animals treated with MSC544-derived control exosomes had reached the criteria for termination of the experiment. Primary tumor tissues from the left and right shoulders were isolated, washed in PBS, and weighted. Bone marrow was harvested by cutting the femur and rinsing the open bone with PBS followed by centrifugation (360× *g*/7 min) of the bone marrow cells. Organs were also dissected from the mice and evaluated by fluorescence microscopy for the presence and accumulation of metastatic cells.

### 4.6. Transcript Analysis by RT-PCR

Total RNA was isolated from the tumor tissues and the organs using the RNeasy Mini Kit (Qiagen, Hilden, Germany) according to the manufacturer’s instructions. An amount of 1 µg of RNA was reverse-transcribed into cDNA, and reactions were performed with corresponding primers (mCherry: sense 5′-TTC ATG TAC GGC TCC AAG GC-3′; antisense 5′-CTG CTT GAT CTC GCC CTT CA-3′; amplification product 297 bp; GAPDH as a control: sense 5′-ACC ACA GTC CAT GCC ATC AC-3′; antisense 5′-TCC ACC ACC CTG TTG CTG TA-3′; amplification product 452 bp) specifically as described previously [27]. Aliquots of 25 µL of each RT-PCR product were separated on a 2% agarose gel including the standard GeneRuler 100 bp DNA Ladder (ThermoFisher Scientific, Schwerte, Germany) and visualized by GelRedTM (Biotium Inc., Hayward, CA, USA) staining.

## 5. Conclusions

In contrast to growth-restricted primary MSC, permanently proliferating MSC544 provide an unlimited source of reproducible properties, including exosome production in high quantities, which are required in regenerative medicine and tumor therapeutic approaches. These MSC544-derived exosomes can be modified or loaded with several chemotherapeutic compounds. Following systemic intravenous application, these vesicles can address tumors and accompanying metastases by reduction of the neoplastic tissues. The efficiency of drug-loaded MSC544-derived exosomes with more focused tumor targeting would correlate with a reduction in side effects. Moreover, loading of MSC544 exosomes with different drugs enables a reproducible delivery to tumors by using the same vehicle system which elevates therapeutic potential. However, use of cell-free vesicles such as exosomes in clinical applications with respect to, for example, biodistribution and biocompatibility, optimized application amount of exosomes, and pharmacokinetics of drug-loaded exosomes still requires various efforts for standardization.

## Figures and Tables

**Table 1:** Analysis of MSC544 microvesicles and exosomes after taxol treatment.

Vesicles	Zeta Potential (mV)	Concentration (Vesicles/mL)
Control exosomes	−30.3	8.8 × 10^11^
Taxol microvesicles	−19.1	2.0 × 10^11^
Taxol exosomes	−15.1	2.8 × 10^11^

## Supplementary Materials

The following are available online at https://www.mdpi.com/1422-0067/21/19/7311/s1. Figure S1: The relative effectiveness of MSC544-derived taxol exosomes on the reduction in tumor volumes was expressed as percentage of control exosomes normalized to 100%. Data of taxol substance were calculated from a previous study with similar experimental conditions [28]. Thereby, taxol data were adjusted by a factor of 4.9 due to differences in the mean volume of control tumors resulting from the application of 2 × 10^6^ MDA-hyb1 cells into one mouse flank in the previous experiment as compared to the present injection of 1 × 10^6^ MDA-hyb1 cells into both flanks, respectively [28].

## Abbreviations

CAFs	carcinoma-associated fibroblasts
EVs	extracellular vesicles
hUC	human umbilical cord
miR	micro RNA
MSC	mesenchymal stroma/stem-like cells
NTA	nanoparticle tracking analysis
TNBC	triple negative breast cancer
TRAIL	tumor necrosis factor-related apoptosis-inducing ligand
