# Supplementary figures and images for: Anti-Tumor Effects of Exosomes Derived from Drug-Incubated Permanently Growing Human MSC
Source: Int J Mol Sci. 2020 Oct 3;21(19):7311. doi: 10.3390/ijms21197311 (PMC7582671; doi:10.3390/ijms21197311)

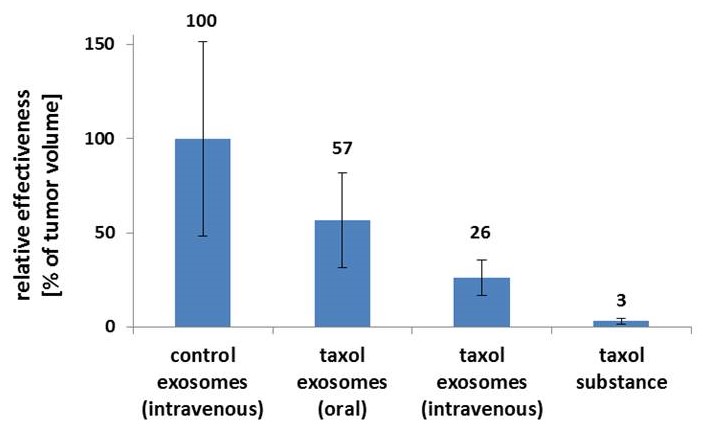

Supplement: Supplementary file 1 [file ijms-21-07311-s001.jpg]
